# LDN-27219 Modulates High-Glucose-Induced Endothelial Bioenergetic Remodeling and MitoSOX Red Fluorescence

**DOI:** 10.3390/life16081214

**Published:** 2026-07-23

**Authors:** Augusta Volkevičiūtė, Deimantė Puzinovė, Zbigniev Balion, Nikolas Žumbakis, Patricija Lapinskaitė, Agilė Tunaitytė, Olena Kutakh, Edgaras Stankevičius, Estéfano Pinilla, Ulf Simonsen

**Affiliations:** 1 Preclinical Research Laboratory for Medicinal Products, Institute of Cardiology, Lithuanian University of Health Sciences, LT-50162 Kaunas, Lithuania; 2 Department of Biomedicine, Pulmonary and Cardiovascular Pharmacology, Aarhus University, DK-8000 Aarhus, Denmark

**Keywords:** LDN-27219, transglutaminase-2, endothelial cells, high glucose, mitochondrial respiration, extracellular acidification rate, MitoSOX Red fluorescence, cellular reducing capacity, endothelial bioenergetics, diabetic vascular dysfunction

## Abstract

Background: Endothelial dysfunction is a key feature of diabetic vascular disease and is associated with high-glucose-induced changes in endothelial energy metabolism and redox balance. Transglutaminase-2 has been implicated in vascular stress responses, but its contribution to endothelial adaptation to high glucose remains incompletely defined. Methods: EA.hy926 endothelial cells were cultured under normoglycaemic (NG) or high-glucose conditions and treated with LDN-27219 at 20 µg/mL (48.9 µM). Cellular reducing capacity, total protein content, mitochondrial respiration, MitoSOX Red fluorescence, and selected cytokine and adhesion-marker secretion were assessed. Results: High glucose shifted cells toward a more glycolytic basal phenotype, increased MitoSOX Red fluorescence, and reduced glycolytic stress responsiveness compared with NG conditions. LDN-27219 had limited effects under NG conditions but, under high-glucose conditions, reduced basal respiration, maximal respiration, ATP-linked oxygen consumption, and spare respiratory capacity, while partially restoring stressed extracellular acidification. LDN-27219 also reduced high-glucose-induced MitoSOX Red fluorescence, although this cannot be interpreted as definitive evidence of reduced mitochondrial superoxide production because mitochondrial membrane potential was not measured. No statistically significant changes were detected in the selected inflammatory or adhesion markers. Conclusions: LDN-27219 modifies endothelial bioenergetic and oxidant-associated fluorescence responses under high-glucose conditions.

## 1. Introduction

Diabetes mellitus is a major risk factor for cardiovascular disease, and vascular complications remain among the principal causes of morbidity and mortality in patients with chronic metabolic disorders. Endothelial dysfunction is an early and central feature of diabetic vascular injury and contributes to impaired vasomotor regulation, increased vascular permeability, inflammatory activation, and progressive microvascular and macrovascular damage [1]. Sustained hyperglycaemia is a major driver of this process, promoting oxidative stress, reducing nitric oxide bioavailability, and disrupting endothelial homeostasis [2].

Mitochondria are increasingly recognized as important regulators of endothelial stress adaptation. Although endothelial cells rely predominantly on glycolysis for ATP generation, mitochondria are essential for redox signaling, calcium handling, apoptotic regulation, metabolic flexibility, and coordination of cellular responses to injury [3]. Under hyperglycaemic conditions, endothelial mitochondria undergo functional and structural remodeling, including altered respiratory behavior, impaired mitochondrial dynamics, disturbed quality-control processes, and increased production of mitochondrial reactive oxygen species (ROS) [4]. These changes are highly relevant to diabetic vascular pathology, as excessive mitochondrial ROS generation can amplify oxidative injury, impair endothelial signaling, and reinforce a maladaptive metabolic state [5].

The molecular mechanisms that connect hyperglycaemia to endothelial mitochondrial remodeling remain incompletely understood. Transglutaminase-2 (TG2) is a multifunctional protein with enzymatic, scaffolding, GTP-binding, and protein-folding-related activities that enable it to participate in diverse cellular stress-response pathways [6]. In addition to its established functions in extracellular matrix remodeling and cell signaling, TG2 has been linked to redox regulation, apoptosis, and mitochondrial biology. TG2 has also been linked to mitochondrial homeostasis and intracellular stress-response pathways, suggesting that it may influence endothelial adaptation to metabolic stress [7,8].

These observations suggest that TG2 may contribute to the endothelial mitochondrial response to metabolic stress. This question is particularly relevant in hyperglycaemia, where oxidative stress, altered bioenergetics, and inflammatory activation develop in parallel and may mutually reinforce endothelial dysfunction. However, despite growing interest in TG2 as a regulator of vascular pathology, its direct contribution to endothelial mitochondrial respiration, glycolytic adaptation, and mitochondrial superoxide generation under high-glucose conditions remains insufficiently defined [6,7,8].

The present study therefore investigated the effect of LDN-27219 on endothelial metabolic and oxidant-associated responses to high-glucose stress. Using EA.hy926 endothelial cells cultured under normoglycaemic and high-glucose conditions, we assessed the impact of LDN-27219 on cellular reducing capacity, cell-associated protein content, mitochondrial respiration, MitoSOX Red fluorescence, and secretion of selected inflammatory and endothelial activation markers. We hypothesized that pharmacological modulation using the TG2 inhibitor LDN-27219 may influence endothelial bioenergetic adaptation and oxidant-associated fluorescence responses under high-glucose conditions.

## 2. Materials and Methods

### 2.1. Cell Culture and Experimental Conditions

Experiments were performed using the human endothelial cell line EA.hy926 obtained from ATCC. Cells were cultured in Dulbecco’s Modified Eagle Medium (DMEM) containing sodium pyruvate at 110 mg/L under two glucose conditions: normoglycaemic, corresponding to 5.5 mM glucose, and high glucose, corresponding to 25 mM glucose. Cells were maintained at 37 °C in a humidified atmosphere containing 5% CO_2_.

For experimental treatments, cells were seeded at assay-specific densities and allowed to attach for 24 h. Subsequently, cells were treated for an additional 24 h with LDN-27219 (MedChemExpress, Monmouth Junction, NJ, USA; catalog no. HY-16693) or vehicle control under the corresponding glucose condition. The final working concentration of LDN-27219 used for Seahorse analysis, MitoSOX Red imaging, and cytokine profiling was 20 µg/mL (48.9 µM). Vehicle-treated cells received DMSO at a final concentration of 0.005%. Cells were not serum-starved before treatment. No osmotic control was included for the high-glucose condition.

### 2.2. PrestoBlue Cellular Reducing Capacity Assay

Cellular reducing capacity was assessed using PrestoBlue Cell Viability Reagent (Thermo Fisher Scientific, Waltham, MA, USA), a resazurin-based assay in which the reducing environment of viable cells converts resazurin to resorufin [9]. EA.hy926 endothelial cells were seeded in 96-well plates at a density of 9000 cells/well and cultured under normoglycaemic or high-glucose conditions. To evaluate concentration-dependent responses to LDN-27219, cells were treated for 24 h with 0.5, 1, 2, 5, 10, 20, 30, or 50 µg/mL LDN-27219, corresponding to 1.22, 2.44, 4.89, 12.23, 24.45, 48.9, 73.35, and 122.25 µM, respectively. Following treatment, PrestoBlue reagent was added according to the manufacturer’s instructions, and cells were incubated for 1.5 h at 37 °C. Absorbance was measured at 570 nm, with 600 nm used as the reference wavelength, using a Varioskan LUX multimode microplate reader (Thermo Fisher Scientific). Total protein content per well was determined using the Bradford assay, and the PrestoBlue reducing-capacity signal was normalized to protein content. Data were expressed relative to the normoglycaemic control group, which was set to 100%. Results are presented as mean ± SD from three independent biological replicates.

### 2.3. Total Protein Quantification by Bradford Assay

Total protein per well was determined using the Bradford assay. Following experimental treatment, cells were lysed with 0.04% Triton X-100 (Sigma-Aldrich, St. Louis, MO, USA). Aliquots of cell lysate were incubated with Bradford reagent according to the assay protocol, and absorbance at 595 nm was measured using a microplate reader. Total protein content was calculated using a bovine serum albumin (Sigma-Aldrich) calibration curve.

Total protein content was used as a cell-associated biomass-related readout in the LDN-27219 dose–response analysis. It was also used to normalize mitochondrial respiration data and MitoSOX Red fluorescence measurements where applicable. Results are presented as mean ± SD from three independent biological replicates.

### 2.4. Mitochondrial Respiration Assessment Using Seahorse XFp Analysis

Mitochondrial respiration and glycolytic activity were assessed using a Seahorse XFp Analyzer (Agilent Technologies, Santa Clara, CA, USA). EA.hy926 endothelial cells were seeded in Seahorse XFp cell culture microplates at a density of 4000 cells/well and maintained under normoglycaemic or high-glucose conditions. Following cell attachment, cells were treated for 24 h with vehicle or LDN-27219 at 20 µg/mL (48.9 µM), under the corresponding glucose condition. Cells were not serum-starved before Seahorse analysis and cell confluence at the time of the assay was visually confirmed before measurement.

Prior to the assay, culture medium was replaced with Seahorse XF assay medium supplemented with 10 mM glucose, 2 mM glutamine, and 1 mM pyruvate. This standardized assay medium was applied to all experimental groups and is consistent with commonly used Seahorse Cell Mito Stress Test substrate conditions. Therefore, Seahorse measurements reflect bioenergetic responses after prior 24 h exposure to normoglycaemic or high-glucose culture conditions under common assay-substrate conditions, rather than real-time measurements during continued exposure to the original culture media. Cells were equilibrated at 37 °C in a non-CO_2_ incubator before measurement. Mitochondrial function was evaluated using the Seahorse XF Cell Mito Stress Test according to the manufacturer’s instructions. Oxygen consumption rate (OCR) and extracellular acidification rate (ECAR) were recorded at baseline and following sequential injection of oligomycin (1 µM), FCCP (2 µM), and rotenone/antimycin A (0.5 µM).

Respiratory parameters, including basal respiration, maximal respiration, proton leak, oligomycin-sensitive ATP-linked OCR, and spare respiratory capacity, were calculated using Seahorse Wave software version 2.6.1 [10]. Cellular energy phenotype and metabolic potential outputs were derived from OCR and ECAR measurements under basal and stressed conditions. Following completion of the assay, total protein content per well was determined using the Bradford assay, and Seahorse data were normalized accordingly.

Results are presented as mean ± SD from three independent biological replicates.

### 2.5. MitoSOX Red Fluorescence Imaging

Mitochondrial oxidant-associated fluorescence was assessed using MitoSOX Red mitochondrial superoxide indicator (Thermo Fisher Scientific). Prior to cell seeding, 96-well plates were coated with 0.1% gelatin (Sigma Aldrich) for 30 min to enhance cell adhesion. EA.hy926 endothelial cells were seeded at a density of 9000 cells/well and cultured under normoglycaemic or high-glucose conditions.

Following treatment with vehicle or LDN-27219 at 20 µg/mL (48.9 µM) for 24 h, cells were incubated with 5 µM MitoSOX Red for 15 min at 37 °C protected from light. After staining, cells were washed three times with PBS. Fluorescence images were acquired using an Olympus APEXVIEW APX100 fluorescence microscope (Evident Corporation, Hachioji, Tokyo, Japan) under identical acquisition settings for all experimental groups. Mitochondrial membrane potential was not assessed in parallel with MitoSOX Red imaging.

Quantitative image analysis was performed using ImageJ software 1.54t (U.S. National Institutes of Health, Bethesda, MD, USA). The same image-analysis procedure was applied to all experimental groups. Fluorescence intensity was quantified, normalized to total protein content per well determined by Bradford assay, and expressed relative to the normoglycaemic control group, which was set to 100%.

Results are presented as mean ± SD from three independent biological replicates.

### 2.6. Multiplex Cytokine and Adhesion-Marker Analysis

Cytokine, chemokine, and adhesion-marker levels in cell culture supernatants were quantified using a 20-plex bead-based immunoassay, ProcartaPlex (Thermo Fisher Scientific), according to the manufacturer’s instructions. EA.hy926 endothelial cells were cultured under normoglycaemic or high-glucose conditions and treated with vehicle or LDN-27219 at 20 µg/mL (48.9 µM) for 24 h.

After treatment, cell culture supernatants were collected and clarified by centrifugation at 16,000× *g* for 10 min at 4 °C. Samples were stored at −80 °C until analysis. Samples were analyzed in technical duplicates.

The multiplex panel included the following analytes: CD62E/E-selectin, CD62P/P-selectin, GM-CSF, ICAM-1, IFNα, IFNγ, IL-1α, IL-1β, IL-4, IL-6, IL-8/CXCL8, IL-10, IL-12p70, IL-13, IL-17A, IP-10/CXCL10, MCP-1/CCL2, MIP-1α/CCL3, MIP-1β/CCL4, and TNFα.

Data acquisition was performed using a Luminex 200 system (Luminex Corporation, Austin, TX, USA). Analyte concentrations were calculated from standard curves generated for each target using xPONENT software version 4.2 (Luminex Corporation, Austin, TX, USA). Only analytes with concentrations above the detection limit were included in further analysis.

Results are presented as mean ± SD from three independent biological replicates.

### 2.7. Statistics

Statistical analyses were performed using GraphPad Prism version 11.0.2 (GraphPad Software, Boston, MA, USA). Data are presented as mean ± standard deviation (SD) from three independent biological replicates, unless otherwise stated. For multiplex cytokine measurements, technical duplicate values were averaged for each biological replicate prior to statistical analysis and evaluation.

Where applicable, data were normalized to the normoglycaemic control group, which was defined as 100%. Differences among experimental groups were assessed using one-way analysis of variance (ANOVA) followed by Fisher’s least significant difference (LSD) post hoc test for pairwise comparisons. Fisher’s least significant difference (LSD) test was selected because the study was hypothesis-driven and involved a limited number of predefined group comparisons. The exploratory nature of the study and the small number of biological replicates were considered when interpreting statistically significant findings. A *p*-value < 0.05 was considered statistically significant.

**Figure 1:**
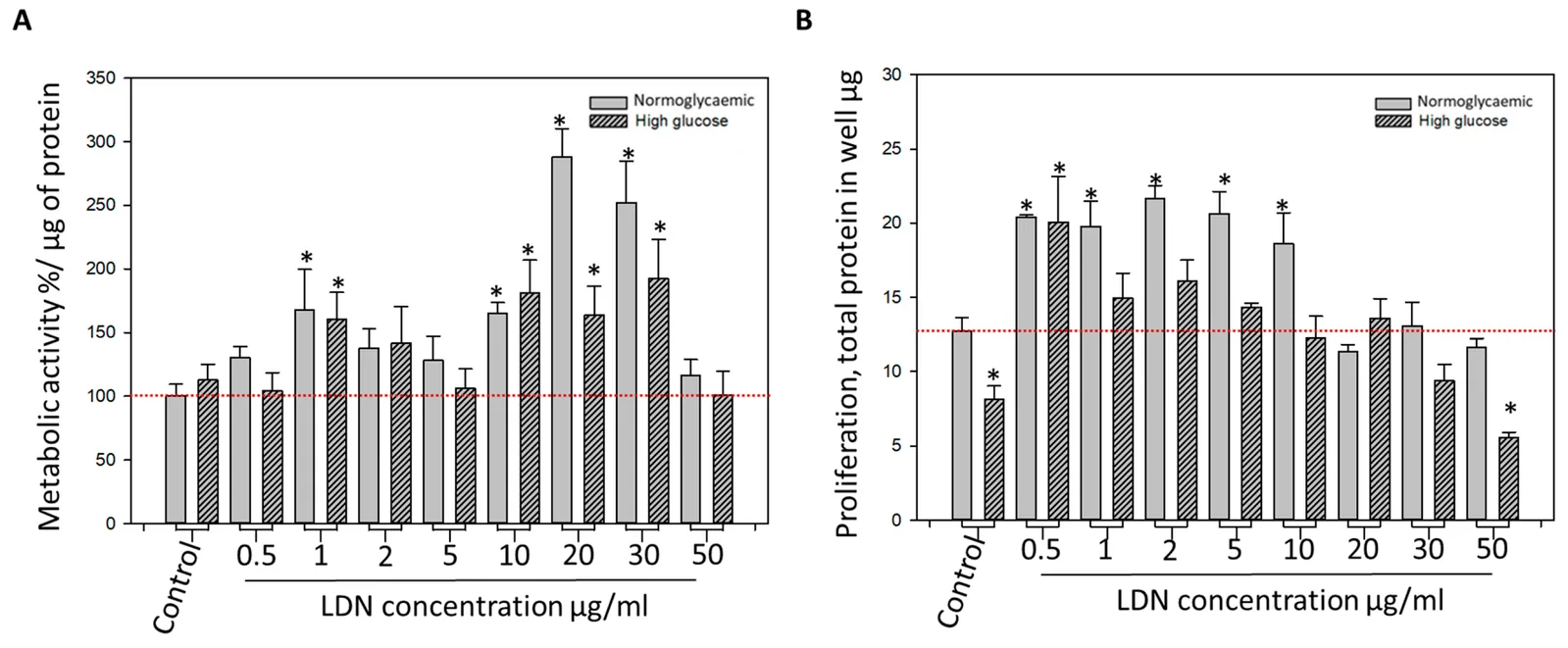
Dose-dependent effects of LDN-27219 on cellular reducing capacity and total protein content under normoglycaemic and high-glucose conditions. EA.hy926 endothelial cells were cultured under normoglycemic or high-glucose conditions and treated for 24 h with increasing concentrations of LDN-27219. (**A**) Cellular reducing capacity was assessed using the PrestoBlue assay, normalized to total protein content, and expressed relative to the normoglycemic control group, which was set to 100%. (**B**) Total protein content per well was determined using the Bradford assay and used as a cell-associated biomass-related readout. Data are presented as mean ± SD from three independent biological replicates. Statistical analysis was performed using one-way ANOVA followed by Fisher’s LSD post hoc test. The red dashed line indicates the normoglycemic control reference level. Significant comparisons are indicated in the figure. * *p* < 0.05 versus the untreated normoglycaemic control.

**Figure 2:**
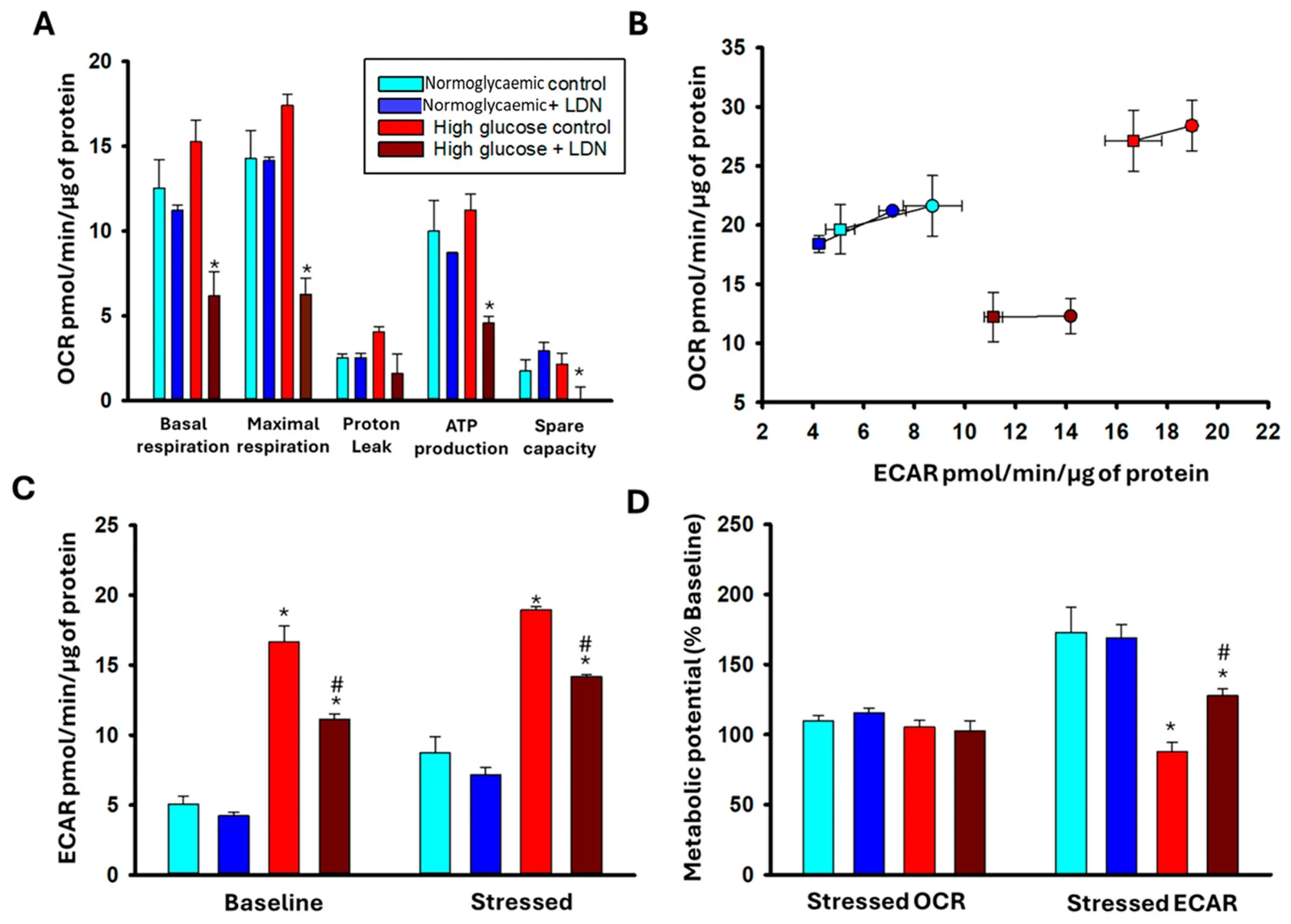
LDN-27219 alters endothelial bioenergetic responses under high-glucose conditions. EA.hy926 endothelial cells were cultured under normoglycemic or high-glucose conditions and treated for 24 h with vehicle or LDN-27219 at 20 µg/mL (48.9 µM). Mitochondrial respiration and glycolytic activity were assessed using the Seahorse XFp Cell Mito Stress Test. (**A**) Quantification of calculated mitochondrial respiratory parameters after subtraction of non-mitochondrial respiration, including basal respiration, maximal respiration, proton leak, ATP-linked OCR, and spare respiratory capacity. (**B**,**C**) Cellular energy phenotype profiles under basal and stressed conditions, showing total OCR, including both mitochondrial and non-mitochondrial OCR, and ECAR relationships across experimental groups. (**D**) Quantification of metabolic potential expressed as stressed total OCR, including both mitochondrial and non-mitochondrial OCR, and stressed ECAR. Data are presented as mean ± SD from three independent biological replicates. Statistical analysis was performed using one-way ANOVA followed by Fisher’s LSD post hoc test. * *p* < 0.05 versus normoglycemic control; # *p* < 0.05 versus high-glucose control.

**Figure 3:**
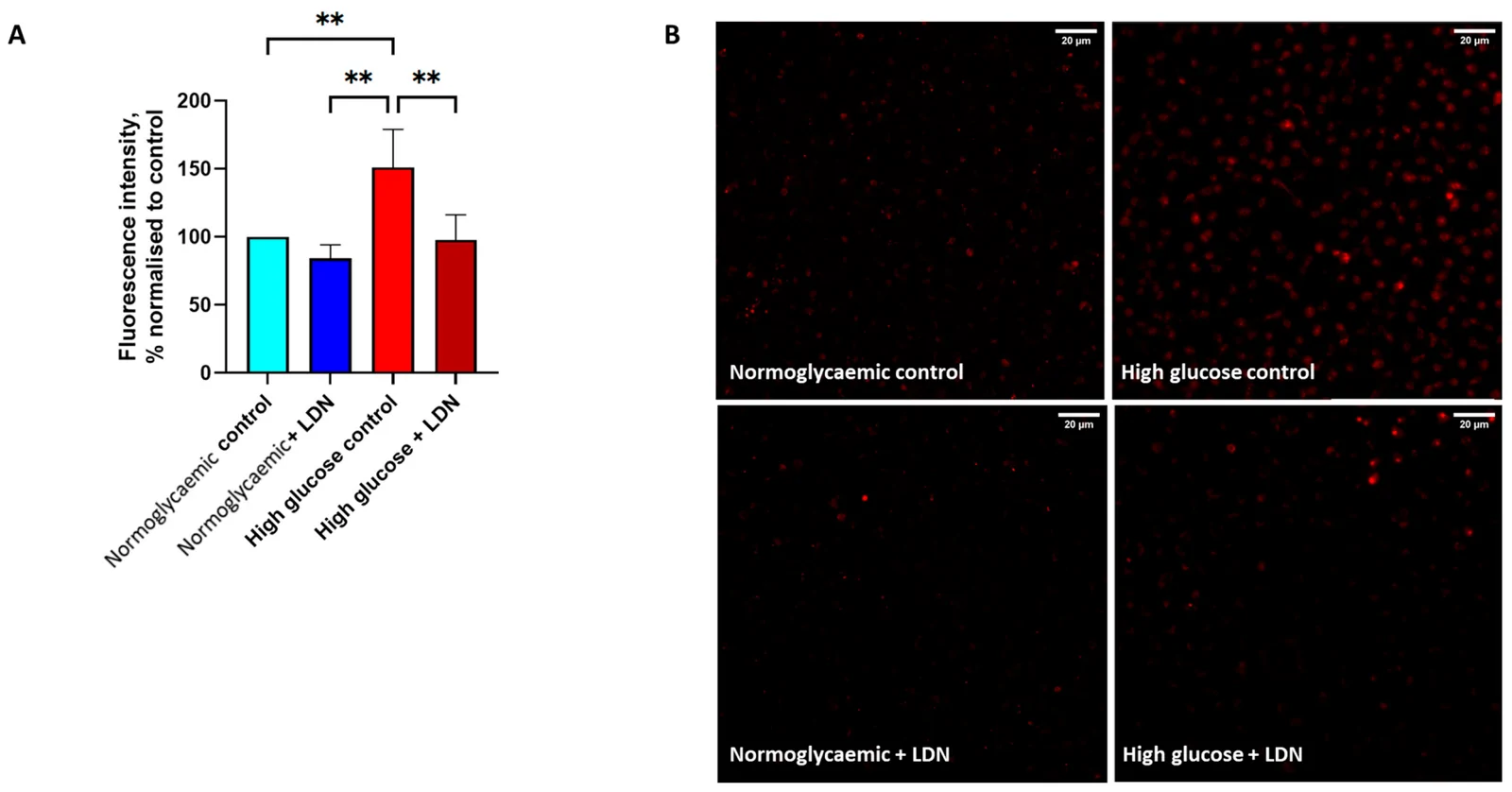
LDN-27219 reduces high-glucose-induced MitoSOX Red fluorescence in endothelial cells. EA.hy926 endothelial cells were cultured under normoglycemic or high-glucose conditions in the absence or presence of LDN-27219 at 48.9 µM, corresponding to 20 µg/mL. Mitochondrial oxidant-associated fluorescence was assessed using MitoSOX Red. (**A**) Quantification of MitoSOX Red fluorescence intensity normalized to total protein content and expressed relative to the normoglycemic control group, which was set to 100%. Data are presented as mean ± SD from 3 independent biological replicates. Statistical analysis was performed using one-way ANOVA followed by Fisher’s LSD post hoc test. ** *p* < 0.01. (**B**) Representative fluorescence images of endothelial cells under the indicated experimental conditions. Scale bar = 20 µm.

**Figure 4:**
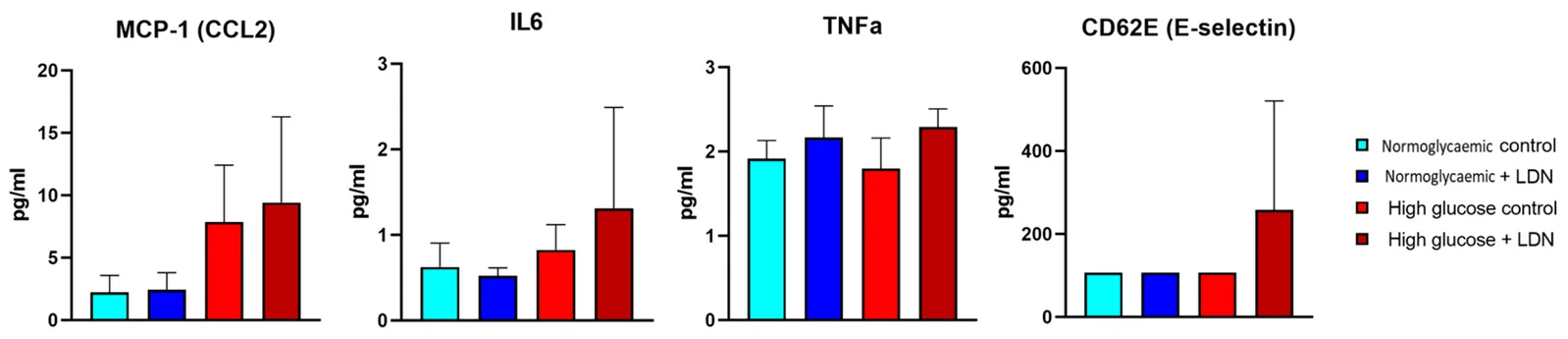
Selected cytokine and adhesion-marker secretion under low- and high-glucose conditions after LDN-27219 treatment. EA.hy926 endothelial cells were cultured under normoglycemic or high-glucose conditions in the absence or presence of LDN-27219 at 20 µg/mL, corresponding to 48.9 µM. Secretion of MCP-1/CCL2, IL-6, TNFα, and CD62E/E-selectin into the culture supernatant was quantified using a multiplex Luminex assay. Data are presented as mean ± SD from three independent biological replicates. Statistical analysis was performed using one-way ANOVA followed by Fisher’s LSD post hoc test. No statistically significant differences were detected among groups for the analytes shown.

In the figures, statistical significance is indicated as defined in the corresponding legends. For Figure 1, * *p* < 0.05 versus the normoglycaemic control group. For Figure 2, * *p* < 0.05 versus the normoglycaemic control group and # *p* < 0.05 versus the high-glucose control group. For Figure 3, ** *p* < 0.01 for the indicated comparisons. No statistically significant differences were detected among groups for the cytokine and adhesion-marker measurements shown in Figure 4.

## 3. Results

### 3.1. Selection of LDN-27219 Concentration Based on Endothelial Cellular Reducing Capacity and Total Protein Content

LDN-27219 induced a concentration-dependent change in normalized cellular reducing capacity under both glucose conditions. In normoglycaemic cells, the PrestoBlue signal increased significantly at 1, 10, 20, and 30 µg/mL LDN-27219 relative to the normoglycaemic control, with the highest response observed at 20 µg/mL. A significant increase was also detected in high-glucose cells treated with 1, 10, 20, and 30 µg/mL LDN-27219. However, the response differed between glucose conditions, with a more pronounced increase at 20 and 30 µg/mL under normoglycaemic conditions. At 50 µg/mL, the PrestoBlue signal returned toward the control range in both glucose conditions, yielding a biphasic concentration–response profile with peak reducing capacity at intermediate concentrations (Figure 1A).

Total protein content displayed a distinct concentration–response pattern. High-glucose control cells showed significantly lower total protein content than normoglycaemic control cells. Under normoglycaemic conditions, LDN-27219 significantly increased total protein content at 0.5, 1, 2, 5, and 10 µg/mL. In contrast, under high-glucose conditions, a significant increase in total protein content was observed only at 0.5 µg/mL, whereas higher concentrations did not produce a comparable increase. At 50 µg/mL, total protein content in high-glucose cells was significantly reduced relative to normoglycaemic control cells, indicating an unfavorable biomass-associated response at the highest concentration tested (Figure 1B).

Based on the combined PrestoBlue and total protein profiles, 20 µg/mL LDN-27219 was selected as the working concentration for subsequent assays because it did not produce overt loss of cell-associated protein content under either glucose condition while remaining within the non-decreasing range of the PrestoBlue dose–response. This concentration corresponds to 48.9 µM and should be interpreted operationally rather than as evidence of optimal cellular health or maximal TG2-specific efficacy.

### 3.2. LDN-27219 Alters High-Glucose-Associated Endothelial Bioenergetic Remodeling

To assess whether LDN-27219 modifies endothelial bioenergetic responses under normoglycaemic and high-glucose conditions, mitochondrial respiration and glycolytic activity were evaluated using Seahorse XFp analysis. LDN-27219 did not markedly alter the overall cellular energy phenotype under normoglycaemic conditions, as reflected by the close overlap between normoglycaemic control and normoglycaemic + LDN-27219 groups in basal and stressed OCR–ECAR profiles (Figure 2B,C).

Respiratory parameters in Figure 2A represent calculated mitochondrial OCR after subtraction of non-mitochondrial respiration, whereas the OCR values in the energy phenotype and metabolic potential plots represent total OCR; therefore, these outputs should be interpreted as related but not numerically identical Seahorse-derived measures.

In contrast, high-glucose cells displayed increased ECAR without a significant increase in OCR relative to normoglycaemic control cells, consistent with a shift toward a more glycolytic basal phenotype rather than a uniformly more energetic phenotype. Treatment with LDN-27219 substantially altered this high-glucose-associated phenotype. Under high-glucose conditions, LDN-27219 reduced basal respiration to approximately half of the normoglycaemic control value, and FCCP failed to elicit a further increase in OCR, indicating loss of spare respiratory capacity. This effect was not observed under normoglycaemic conditions, where basal respiration and spare capacity were preserved, suggesting that the suppression of mitochondrial respiration by LDN-27219 was glucose-context-dependent (Figure 2A–C).

In high-glucose cells, LDN-27219 reduced OCR across the Mito Stress Test profile: basal respiration decreased, FCCP-stimulated respiration did not exceed basal values, and oligomycin-sensitive ATP-linked OCR was correspondingly reduced. The proton leak was not increased, indicating that the reduction in OCR was not explained by enhanced uncoupled respiration. However, ATP-linked OCR should not be interpreted as a direct measurement of total cellular ATP content. The Seahorse data cannot determine whether total ATP abundance was reduced or maintained through compensatory glycolytic activity; direct ATP measurement would be required to resolve this (Figure 2A).

Analysis of stressed metabolic responses revealed glucose- and treatment-dependent effects on glycolytic adaptation. High-glucose control cells showed significantly reduced stressed ECAR compared with normoglycaemic groups, indicating that the elevated basal glycolytic phenotype was associated with a compressed glycolytic stress response. In high-glucose + LDN-27219 cells, stressed ECAR was significantly higher than in high-glucose control cells, indicating partial recovery of the glycolytic stress response under high-glucose conditions, although values did not reach those observed in normoglycaemic groups. Stressed OCR did not differ significantly between groups in this analysis (Figure 2D). Taken together, these findings suggest that high-glucose exposure shifts EA.hy926 cells toward a more glycolytic basal phenotype. LDN-27219 markedly alters this phenotype under high-glucose conditions by suppressing mitochondrial respiratory output, eliminating spare respiratory capacity, reducing ATP-linked OCR, and partially restoring stressed ECAR.

### 3.3. LDN-27219 Reduces High-Glucose-Induced MitoSOX Red Fluorescence

To determine whether high-glucose-associated bioenergetic remodeling was accompanied by changes in mitochondrial oxidant-associated fluorescence, cells were stained with MitoSOX Red. High-glucose exposure markedly increased MitoSOX Red fluorescence compared with normoglycaemic control cells. A similar difference was observed between high-glucose control cells and normoglycaemic cells treated with LDN-27219, indicating that the increased fluorescence signal was associated primarily with the high-glucose condition rather than with LDN-27219 exposure alone.

Treatment with LDN-27219 significantly reduced the high-glucose-induced MitoSOX Red signal. In contrast, LDN-27219 did not increase MitoSOX Red fluorescence under normoglycaemic conditions, where fluorescence remained comparable to or slightly below the normoglycaemic control level. These findings indicate that high glucose increased an oxidant-associated mitochondrial fluorescence signal that was reduced by LDN-27219. However, because MitoSOX Red accumulation is influenced by mitochondrial membrane potential, these data should not be interpreted as definitive evidence of reduced mitochondrial superoxide production without parallel membrane-potential measurements.

Representative fluorescence microscopy images illustrate the MitoSOX Red fluorescence patterns observed across the experimental groups and used for quantitative image analysis. High-glucose control cells displayed a visibly stronger MitoSOX Red signal than normoglycaemic control cells, consistent with the higher fluorescence intensity quantified in Figure 3A. This fluorescence pattern was reduced following LDN-27219 treatment under high-glucose conditions, whereas normoglycaemic cells treated with LDN-27219 maintained a comparatively weak fluorescence signal.

### 3.4. Soluble Inflammatory and Endothelial Activation-Associated Mediator Profiles After LDN-27219 Treatment

To examine whether the metabolic and mitochondrial effects of LDN-27219 were accompanied by changes in endothelial inflammatory signaling, the concentrations of selected cytokines, chemokines, and adhesion-related mediators were assessed in cell culture supernatants using a multiplex Luminex assay.

MCP-1/CCL2 secretion was low under normoglycaemic conditions and appeared higher in both high-glucose groups. A similar tendency was observed for IL-6, with modestly increased mean concentrations in high-glucose control cells and a further numerical increase in high-glucose cells treated with LDN-27219. However, due to the variability within these groups, neither MCP-1 nor IL-6 differed significantly between experimental conditions (Figure 4).

TNFα concentrations remained comparable across all groups, with only minor variation between normoglycaemic and high-glucose conditions and no evident effect of LDN-27219 treatment. CD62E/E-selectin levels were also broadly similar among normoglycaemic control, normoglycaemic + LDN, and high-glucose control cells. Although the high-glucose + LDN group showed a numerically higher mean CD62E concentration, this response was highly variable and did not reach statistical significance.

Overall, no statistically significant differences were detected among groups for MCP-1/CCL2, IL-6, TNFα, or CD62E/E-selectin under the present experimental conditions. Given the small number of biological replicates and the variability observed in several analytes, these data should be interpreted as exploratory and do not exclude more subtle or delayed effects of high glucose or LDN-27219 on endothelial inflammatory signaling.

## 4. Discussion

The present study shows that LDN-27219 modifies endothelial adaptation to high-glucose stress at the level of cellular reducing capacity, mitochondrial bioenergetics, and MitoSOX Red fluorescence. Diabetes-associated endothelial dysfunction is increasingly understood as a multifactorial process in which hyperglycemia, oxidative stress, mitochondrial remodeling, and inflammatory signaling interact to promote vascular injury and loss of endothelial homeostasis [1,2,3,4,5]. Within this framework, the current findings suggest that pharmacological modulation with LDN-27219 alters endothelial metabolic responses to high-glucose exposure. However, because LDN-27219 was used at a supra-EC50 concentration and TG2 target engagement was not directly assessed, the findings should be interpreted as LDN-27219-associated effects rather than definitive evidence of TG2-specific signaling [6,11,12]. The dose–response analysis was used to identify an operational working concentration for subsequent mechanistic experiments. Lower concentrations of LDN-27219 were included in the initial concentration–response experiment, ranging from 0.5 to 10 µg/mL, but 20 µg/mL was selected because it did not produce overt loss of cell-associated protein content under either glucose condition while remaining within the non-decreasing portion of the PrestoBlue reducing-capacity response. Importantly, this selection criterion should not be interpreted as evidence that 20 µg/mL (48.9 µM) is biologically optimal, protective, or maximally TG2-specific. LDN-27219 has been described as a potent, slow-binding, reversible TG2 inhibitor with a reported IC_50_ of 0.6 µM [11]; therefore, the concentration used in the present study, 20 µg/mL (48.9 µM), is substantially above its reported inhibitory potency and may increase the possibility of TG2-independent or off-target effects [9]. Previous experimental studies investigating the vascular effects of LDN-27219 have also employed concentrations in the low- to mid-micromolar range to achieve consistent biological responses in isolated tissues and cell-based systems [11,12]. Future studies should test lower concentrations, particularly in the low-micromolar range, and include direct TG2 activity or target-engagement assays. A central observation of the present study was that high-glucose exposure shifted endothelial cells toward a more glycolytic basal phenotype, reflected by increased ECAR without a significant increase in OCR. This distinction is important because the data do not support the conclusion that high glucose uniformly increase cellular energetic activity. Previous studies have shown that high-glucose exposure can induce endothelial metabolic remodeling, including altered mitochondrial respiration, ATP handling, mitochondrial ultrastructure, and oxidative stress burden [13,14,15,16]. However, hyperglycaemia does not produce a single fixed bioenergetic phenotype across endothelial models; responses vary according to cell type, exposure duration, substrate conditions, and analytical endpoint [5,17,18]. The present findings should therefore be interpreted as evidence of glucose-dependent bioenergetic remodeling rather than generalized enhancement of mitochondrial function [5,16,17,18]. LDN-27219 had little apparent effect on the cellular energy phenotype under normoglycaemic conditions, whereas its effect under high-glucose conditions was pronounced. In high-glucose cells, LDN-27219 significantly decreased basal respiration, maximal respiration, ATP-linked respiration, and spare respiratory capacity, while basal respiration approached maximal respiration, indicating that virtually no reserve respiratory capacity remained available after metabolic stress. Interpretation of these Seahorse-derived parameters requires consideration of the full respiratory profile rather than evaluation of single indices in isolation [10]. In this context, the parallel reduction in ATP-linked respiration and spare respiratory capacity indicates that LDN-27219 strongly constrained oxidative metabolic output specifically in the high-glucose environment.

The stressed ECAR data add further nuance to this interpretation. High-glucose control cells displayed reduced glycolytic metabolic potential under stress despite their more glycolytic basal state, whereas LDN-27219 partially restored stressed ECAR despite markedly reducing OCR. This suggests that LDN-27219 may shift high-glucose endothelial cells away from a respiration-dependent stress response while partially preserving glycolytic adaptability under challenge [16].

It is important to note that an osmotic control, such as mannitol or L-glucose, was not included in our model for the cells in normoglycaemic conditions, and we therefore address the potential contribution of hyperosmolarity to the glucose-associated effects reported here. Two aspects of the experimental design constrain this concern. First, the difference in osmolarity between the normoglycaemic and high-glucose conditions, approximately 19.5 mOsm, was present only during the 24 h preconditioning period; all groups were subsequently transferred to the manufacturer-recommended assay medium containing 10 mM glucose for the Seahorse measurements. This standard concentration was chosen because the concentration of glucose in the assay medium modulates the balance between glycolytic and oxidative ATP production, and standardized substrate conditions are required for interpretable extracellular flux data [10]. As all groups were measured under identical osmolarity, acute osmotic effects cannot account for the differences observed, and only an adaptation-based osmotic effect imprinted during preconditioning could plausibly persist into the assay. Second, the effects of LDN-27219, which constitute the principal findings of this study, were assessed as a within-high-glucose comparison, high-glucose control versus high-glucose + LDN-27219; these groups were preconditioned and measured under identical osmolarity. Therefore, osmotic differences are less likely to explain the within-high-glucose effects of LDN-27219, although osmotic adaptation during preconditioning cannot be fully excluded.

The remaining question, therefore, is whether chronic hyperosmolar adaptation contributes to the glycolytic shift observed at baseline between normoglycaemic and high-glucose cells. The most severe consequences of osmotic stress, including apoptosis and DNA damage, are characterized at levels of osmotic pressure one order of magnitude higher than those used in our experiment, approximately +200 mOsm and above [17]. Nonetheless, milder hyperosmolarity comparable to that in our hyperglycaemic model has been shown to increase endothelial ROS and inflammatory markers [18] and, in epithelial cells, altered cellular bioenergetics [19]. Therefore, we cannot entirely exclude an osmotic contribution to the baseline glycolytic phenotype observed here. However, we consider it unlikely that osmotic effects fully account for these changes, since our findings of elevated basal ECAR without increased OCR are consistent with previous observations in endothelial cells exposed to chronic hyperglycaemia, where an osmolarity-matched L-glucose control did not alter endothelial OCR or ECAR [20].

The reduction in MitoSOX Red fluorescence after LDN-27219 treatment must be interpreted cautiously. In high-glucose cells, LDN-27219 markedly suppressed mitochondrial respiration, reduced ATP-linked OCR, and abolished spare respiratory capacity. In parallel, MitoSOX Red fluorescence was reduced. This pattern could reflect lower mitochondrial oxidant formation, reduced mitochondrial dye accumulation due to altered membrane potential, or a combination of both. Because mitochondrial membrane potential was not measured, the present data cannot distinguish among these possibilities. Thus, the MitoSOX Red result should be interpreted as a change in mitochondrial oxidant-associated fluorescence rather than definitive evidence of reduced mitochondrial superoxide production [21,22]. Excessive mitochondrial ROS generation remains highly relevant to diabetic endothelial dysfunction, but the current dataset does not allow a firm conclusion that LDN-27219 reduced mitochondrial superoxide generation independently of changes in mitochondrial membrane potential [2,3,4,5,23]. The mitochondrial findings are highly relevant in the context of current TG2 biology. TG2 has been implicated in extracellular matrix remodeling, redox-sensitive signaling, endothelial permeability, diabetic vascular complications, and microvascular injury [6,23,24,25]. In particular, TG2-dependent regulation of AMPK and GAPDH has recently been shown to contribute to diabetic retinal microvascular leakage, directly linking TG2 activity to hyperglycaemia-driven endothelial injury [25]. Furthermore, pharmacological TG2 modulation with LDN-27219 has been shown to improve vascular relaxation in resistance arteries and to influence endothelial function in human diabetic and non-diabetic vascular tissue [11,12]. The current cellular results complement those vascular observations by suggesting that LDN-27219 can alter endothelial bioenergetic behavior under high-glucose conditions, although direct TG2 target engagement was not assessed in the present study.

In contrast to the marked effects on mitochondrial respiration and MitoSOX Red fluorescence, LDN-27219 did not significantly alter secretion of MCP-1/CCL2, IL-6, TNFα, or CD62E/E-selectin under the conditions tested. MCP-1 and IL-6 showed numerical elevations under high-glucose conditions, whereas TNFα and CD62E remained broadly unchanged across experimental groups. These findings suggest that, within the 24 h treatment window, mitochondrial metabolic remodeling and reduced MitoSOX Red fluorescence occurred without a robust shift in the selected soluble inflammatory mediators. This does not negate the inflammatory relevance of TG2, since endothelial metabolism and inflammation are tightly interconnected yet may evolve with different kinetics, amplitudes, and endpoint sensitivity depending on the experimental model [26,27,28].

The current cytokine findings should therefore be interpreted cautiously. They indicate that the dominant detectable effects of LDN-27219 in this model were mitochondrial and metabolic rather than strongly secretory-inflammatory. Recent literature highlights that acute and chronic hyperglycaemic stress can exert distinct effects on endothelial function and that the magnitude of endothelial inflammatory output depends heavily on duration of exposure, vascular context, and readout selection [29,30]. Consequently, the absence of statistically significant cytokine changes in the present study does not exclude a role for TG2 in inflammatory endothelial remodeling at later time points or at the level of transcriptional or membrane-bound activation markers.

The present findings remain potentially relevant to diabetic endothelial dysfunction, but their translational interpretation should be approached cautiously [31]. LDN-27219 has been reported to modulate vascular tone and endothelial function in resistance arteries, supporting the broader relevance of TG2-associated pharmacology in vascular biology [11,12]. However, the present study was performed in a single endothelial cell line, used a relatively high LDN-27219 concentration, and did not include direct TG2 activity measurements. Therefore, these data should be viewed as hypothesis-generating evidence that LDN-27219 can alter endothelial bioenergetic adaptation under high-glucose conditions, rather than as proof of a therapeutically beneficial TG2-specific mechanism.

Several limitations must be considered when interpreting the present findings. First, EA.hy926 cells provide a practical and reproducible endothelial model but do not fully reproduce the phenotype of primary vascular endothelial cells or intact vascular tissue. Second, no osmotic control was included for the high-glucose condition; therefore, differences between normoglycaemic and high-glucose groups cannot be attributed exclusively to glucose-specific metabolic signaling and may partly reflect osmolarity-dependent effects. Third, Seahorse measurements were performed in standardized assay medium rather than in the original culture media, and therefore reflect bioenergetic responses after prior glucose-condition exposure under common assay conditions. Fourth, the study relied on pharmacological treatment with LDN-27219 at 20 µg/mL (48.9 µM), a concentration above reported TG2 inhibitory potency ranges; therefore, TG2-independent effects cannot be excluded. Fifth, TG2 expression, transamidase activity, and conformational state were not directly measured, preventing confirmation of target engagement under the present conditions. Sixth, mitochondrial membrane potential was not assessed in parallel with MitoSOX Red fluorescence. Because MitoSOX Red accumulation is influenced by membrane potential, reduced fluorescence in the high-glucose + LDN-27219 group could reflect reduced oxidant formation, reduced dye accumulation, or both. Future validation should combine MitoSOX Red fluorescence with mitochondrial membrane-potential measurements, such as tetramethylrhodamine methyl ester, JC-1, or appropriate mitochondrial mass normalization, together with complementary redox assays. Seventh, Seahorse and MitoSOX Red outputs were normalized to total protein content, but cell number or DNA content was not independently quantified. Cytokine concentrations were reported in culture supernatants and were not additionally normalized to cell-associated biomass. Finally, the experiments were performed with three independent biological replicates, which limits statistical power, particularly for Seahorse and multiplex cytokine analyses. Consequently, both statistically significant and non-significant findings should be interpreted cautiously and confirmed in larger independent studies. In conclusion, LDN-27219 modifies endothelial bioenergetic responses under high-glucose conditions. The treatment suppressed mitochondrial respiratory output, reduced ATP-linked OCR and spare respiratory capacity, and partially recovered stressed ECAR. LDN-27219 also reduced high-glucose-induced MitoSOX Red fluorescence; however, this finding cannot be interpreted as definitive evidence of reduced mitochondrial superoxide production without mitochondrial membrane potential assessment. Overall, these data suggest that LDN-27219 alters endothelial metabolic adaptation under hyperglycaemic conditions. Further studies using lower and mechanistically validated concentrations, direct TG2 target-engagement assays, mitochondrial membrane-potential measurements, ATP quantification, and primary endothelial or vascular models are required to establish TG2-specific mechanisms [23].

## 5. Conclusions

The present study demonstrates that LDN-27219 modifies endothelial bioenergetic responses to high-glucose stress. In EA.hy926 cells, high-glucose exposure was associated with a shift toward a more glycolytic basal phenotype, reduced glycolytic stress responsiveness, and increased MitoSOX Red fluorescence. LDN-27219 markedly altered this response by suppressing mitochondrial respiration, reducing ATP-linked OCR and spare respiratory capacity, and partially recovering stressed ECAR under high-glucose conditions.

LDN-27219 also reduced the high-glucose-induced MitoSOX Red signal. However, because mitochondrial membrane potential was not measured, this finding should be interpreted as a reduction in mitochondrial oxidant-associated fluorescence rather than definitive evidence of decreased mitochondrial superoxide production. No statistically significant changes were detected in the selected soluble inflammatory or endothelial activation markers.

Overall, these data suggest that LDN-27219 alters endothelial metabolic adaptation under hyperglycaemic conditions. Future studies should include lower and mechanistically validated LDN-27219 concentrations, direct TG2 activity or target-engagement measurements, mitochondrial membrane potential assessment, ATP quantification, and validation in primary endothelial cells or vascular tissue.

## Data Availability

The data supporting the findings of this study are available from the corresponding author upon reasonable request. The raw datasets generated during the study, including cellular reducing-capacity measurements, total protein quantification, Seahorse extracellular flux data, MitoSOX Red fluorescence image analysis, and multiplex cytokine measurements, are not publicly archived.

## Abbreviations

The following abbreviations are used in this manuscript:

ANOVA	analysis of variance
ATP	adenosine triphosphate
CCL2	C-C motif chemokine ligand 2
CCL3	C-C motif chemokine ligand 3
CCL4	C-C motif chemokine ligand 4
CD62E	E-selectin
CD62P	P-selectin
CXCL8	C-X-C motif chemokine ligand 8
CXCL10	C-X-C motif chemokine ligand 10
DMEM	Dulbecco’s Modified Eagle Medium
DMSO	dimethyl sulfoxide
ECAR	extracellular acidification rate
FCCP	carbonyl cyanide-p-trifluoromethoxyphenylhydrazone
GM-CSF	Granulocyte–macrophage colony-stimulating factor
ICAM -1	intercellular adhesion molecule 1
IFNα	interferon alpha
IFNγ	interferon gamma
IL	interleukin
LDN	LDN-27219
LSD	least significant difference
MCP-1	monocyte chemoattractant protein-1
MIP-1α	macrophage inflammatory protein-1 alpha
MIP-1β	macrophage inflammatory protein-1 beta
OCR	oxygen consumption rate
PBS	phosphate-buffered saline
ROS	reactive oxygen species
SD	standard deviation
TG2	transglutaminase-2
TNFα	tumor necrosis factor alpha
